# Determinizing the contributions of human activities and climate change on greening in the Beijing–Tianjin–Hebei Region, China

**DOI:** 10.1038/s41598-021-00788-4

**Published:** 2021-10-27

**Authors:** Wei Cao, Dan Wu, Lin Huang, Mei Pan, Taoli Huhe

**Affiliations:** 1 Key Laboratory of Land Surface Pattern and Simulation, Institute of Geographic Sciences and Natural Resources Research, Chinese Academy of Sciences, Beijing, 100101 China; 2 Institute of Urban and Rural Mining, Changzhou University, Changzhou, 213164 China

**Keywords:** Climate-change ecology, Climate-change impacts

## Abstract

China accounts for 25% of the global greening. There are temporal and spatial differences of China’s greening and intrinsic driving forces. Thus, it is crucial to determinize the contributions of human activities and climate change on greening at region scale. The Beijing–Tianjin–Hebei Region (BTHR) is one of the most active areas with human activities in China. It is necessary to explore negative or positive impacts of human activities on the regional greening or browning under climate change. A time series of annual vegetation coverage from satellite data was selected to quantify regional greening in the BTHR from 2000 to 2019 and their responses to climate change and human activities. Results showed generally widespread greening over the last 20 years at an average increased rate of 0.036 decade^−1^ in vegetation coverage (*P* < 0.01). Overall warmer and wetter climate across the BTHR were positively correlated with regional greening. The positive effects of human activities on greening accounted for 48.4% of the BTHR, especially the benefits of ecological restoration projects and the agricultural activities. Increases in vegetation coverage had resulted from the combined effects of climate change and human activities. Climate change had a stronger influence on vegetation coverage than human activities. Contributions of climate change to greening and browning was about 74.1% and < 20%, respectively. The decrease in vegetation coverage was mainly the results of the inhibition of human activities. More detailed socioeconomic and anthropogenic datasets are required for further analysis. Further research consideration would focus on the nonlinear responses of vegetation to climate change.

## Introduction

Vegetation is an important component of terrestrial ecosystems connecting energy flows and matter cycles between the atmosphere and the soil^1^. Greenness is also an important indicator of changes in the global climate and environmental state^2,3^. Long-term satellite records have revealed a significant global greening of vegetated areas since the 1980s^4^. The drivers of global greening include increases in atmospheric CO_2_ concentrations, climate change, nitrogen deposition, and land use change^5^. Studies based on high and medium-resolution satellite data have shown that land use activities including afforestation and the intensification of agriculture have also played significant roles in increasing the vegetation greenness^4^. In a report by the National Aeronautics and Space Administration (NASA), it was noted that China accounted for 25% of the global net increase in leaf area from 2000 to 2017, mainly as a result of afforestation and agricultural activities^6^. These studies have revealed the characteristics and causes of changes in greening at a global and national level; however, greening and its drivers vary significantly at a regional level due to environmental differences and socioeconomic development^7–9^. To implement ecological conservation and rehabilitation measurements, it is necessary to understand the spatiotemporal patterns and drivers of greening at a regional scale^10^.

The Geodetector and residual analysis are among the most widely used methods for analysing and identifying driving factors. The Geodetector is a statistical tool that measures spatial stratified heterogeneity based on spatial variance analysis to quantify the explanatory power of different factors and the relative strengths of their interactions^11–13^. However, there is some subjectivity in the selection of model factors and their classification, and the use of single-year factors or multi-year averages to simulate vegetation index can lead to underestimations of vegetation index changes due to human activities^14^. In the residual analysis method, the effects of human activities are indirectly estimated by simulating changes in vegetation index without human interference in comparison to true vegetation index. This is a simple and effective method for isolating the impacts of human activities on vegetation index from those of climate factors^15,16^. This method has been widely used to quantify the positive and negative effects of human activities on ecosystems based on accessible remote-sensing data. Teng et al. estimated the impacts of climate changes and human activities on net primary productivity vary across an ecotone zone in Northwest China^17^. Shi et al. applied the residual trend analysis to separate impacts of climate change and human activities on vegetation NDVI in the Loess Plateau during the period from 2000 to 2016^18^. Peng et al. analysed dominant drivers on LAI dynamics in karst region of southwest China from 1999 to 2015^19^. In addition, Xu et al. proposed a method for analysing these relative contributions based on residual analysis and quantified the relative contributions of climate change and human activities to the desertification of the Ordos region^20^. Sun et al. and Liu et al. also showed that this method is suitable for evaluating the relative contributions of different factors on vegetation coverage changes in northern China^21,22^.

The Beijing–Tianjin–Hebei region (BTHR) is located in the transition zone between the Inner Mongolian Plateau and the North China Plain, which is one of the most active areas with human activities in China. Also, it plays an important role in the ecological security of North China. Overall, vegetation coverage of the BTHR increased between 2000 and 2015^23,24^. In previous studies of the drivers of vegetation coverage changes in the BTHR, dynamic attribution analyses were not performed and the factors that affect vegetation coverage were not adequately determined^25^. This may have led to underestimations of the impacts of human activities^26^. Against the backdrop of global climate change, the intensification of human socioeconomic activities, and the loss of habitat and biodiversity due to the expansion of production and living space, there is a need to quantify the spatiotemporal characteristics of greening in the BTHR in response to climate change and human activities. Here, trend analysis, correlation analysis, residual analysis, and relative contribution analysis were used to quantify the spatial–temporal variations of greening responded by vegetation coverage of the BTHR between 2000 and 2019 based on the Moderate Resolution Imaging Spectroradiometer (MODIS) products, spatially interpolated meteorological station observations, and socioeconomic statistics. The mechanisms underlying greening responses to climate change and human activities were also investigated. Besides, distinguishing effects of land use changes (grain for green and urban expansion) on regional greening were also analysed. Overall, findings of this study serve as a scientific reference for the conservation and restoration of regional ecosystems.

## Data and methods

### Study area

**Figure 1:**
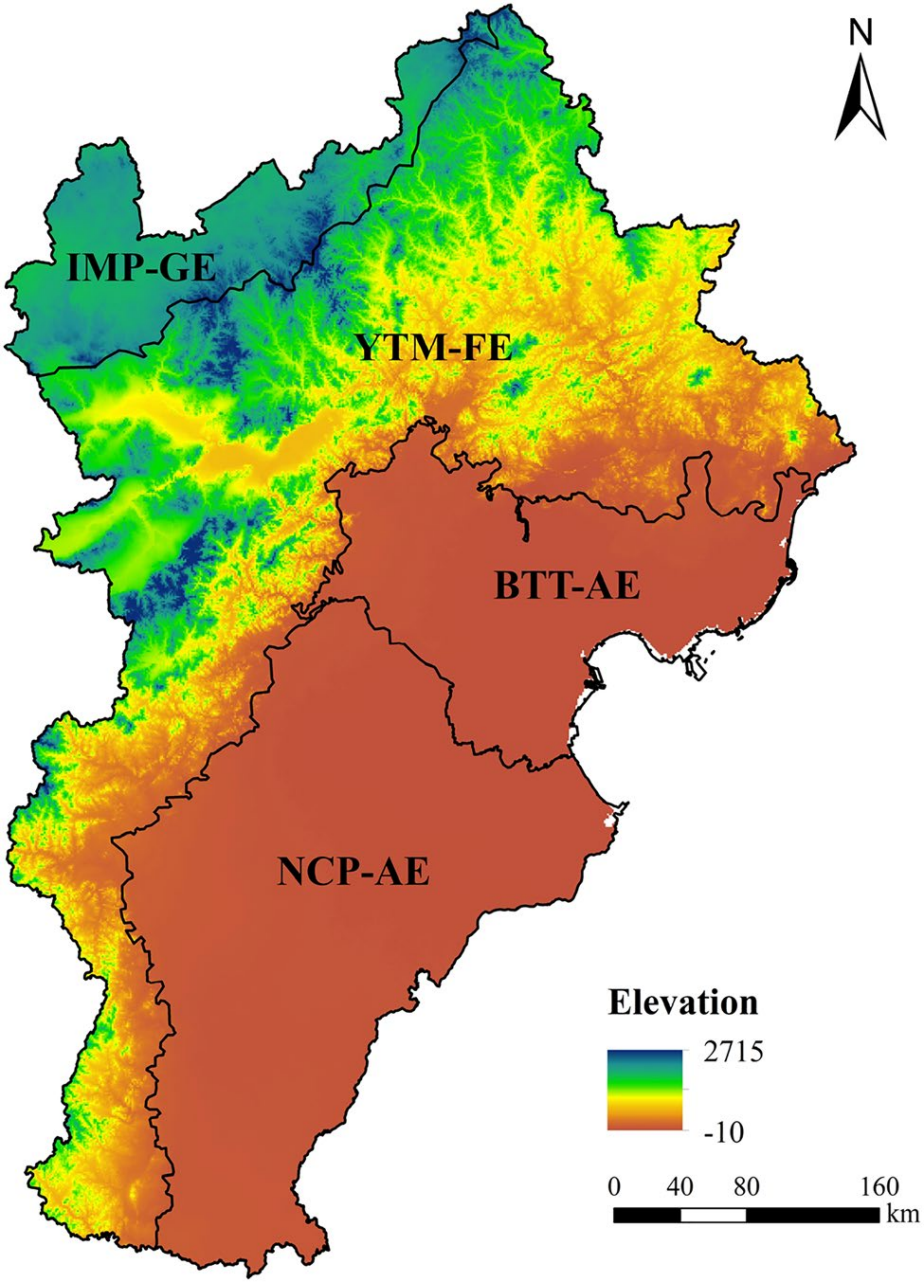
The sub-regional distribution of the study area, with the base map superimposed on the elevation. Map created in ArcMap 10.4.

The BTHR covers an area of 21.6 × 10^4^ km^2^ adjacent to the Inner Mongolian Plateau, the North China Plain, the Taihang Mountains, and the Bohai Bay to the north, south, west, and east, respectively. The terrain of the BTHR is relatively elevated in the northwest and low in the southeast, encompassing a vast array of landscapes including plains, mountains, plateaus, and seas. The central and southern parts of the BTHR are dominated by plains while the northern and western areas are largely covered by highland grasslands, mountains, and fluvial basins. The BTHR has a warm temperate continental monsoon climate characterised by dry and windy spring and autumn seasons, hot and wet summers, and cold and dry winters. The natural vegetation cover in the BTHR mainly consists of sparse deciduous broad-leaved forests and shrubs, with much of this cover being located in the Yanshan Mountains and Taihang Mountains. According to the ecoregion classification criteria of China, the BTHR can be divided into the following four ecoregions from north to south: (1) the grassland ecoregion of the Inner Mongolian Plateau (IMP-GE); (2) the mountainous deciduous broad-leaved forest ecoregion in the Yanshan Mountains and Taihang Mountains (YTM-FE); (3) the agricultural ecoregion in the towns and suburbs of Beijing, Tianjin, and Tangshan (BTT-AE); and (4) the agricultural ecoregion of the North China Plains (NCP-AE) (Fig. 1). The forest ecoregion is the largest of these ecoregions accounting for 49.60% of the total BTHR area. In contrast, the northernmost grassland ecoregion only accounts for 8.24% of the total BTHR area.

### Data sources and processing

The gridded 16-day and 250 m Normalized differential vegetation index (NDVI) MOD13Q1 products were obtained from NASA EOS DATA (https://modis.gsfc.nasa.gov/data/). These data products were then processed by resampling, Savitzky-Golay filtering^27^, and dimidiate pixel model method^28^ to generate the vegetation coverage dataset for the BTHR. Daily precipitation and temperature data were downloaded from the China Meteorological Data Service Centre (http://data.cma.cn/). The ANUSPLIN software was used to interpolate these data to produce monthly and annual precipitation and temperature gridded datasets with a spatial resolution of 1 km.

Global land cover data products (2000, 2020) with a 30-m resolution were obtained from GlobeLand30 (http://www.globallandcover.com/home_en.html), including 10 land cover classes in total, namely cultivated land, forest, grassland, shrubland, wetland, water bodies, tundra, artificial surface, bare land, perennial snow and ice^29^.

Raster data with different resolutions were resampled into cells with the same resolution of 1 km. As socioeconomic indicators, the area of afforestation, total grain production, population density, and gross regional product were obtained from the 2000–2019 editions of the China Statistical Yearbook (http://www.stats.gov.cn/tjsj/ndsj/) and the Hebei Economic Yearbook (http://tjj.hebei.gov.cn/hetj/tjsj/jjnj/).

### Research methods

#### Trend analysis

The Sen’s nonparametric method was used to estimate the slope of the trends in vegetation coverage while the Mann–Kendall method was used to test the significance of the trends^30,31^. The Sen’s equation is as follows:

1 $$ \beta = {\text{Median}}\left( {\frac{{I_{j} - I_{i} }}{j - i}} \right),\;\forall j > i $$

where ‘Median’ is the median; *i* and *j* are time indices; *I*_*i*_ and *I*_*j*_ are the index values of the *i*-th and *j*-th time series, respectively; and *β* > 0 is indicative of a rising trend while *β* < 0 is indicative of a downwards trend. The trends were further categorised into the following categories: insignificant increase (*β* ≥ 0, *P* > 0.05); significant increase (*β* ≥ 0, 0.01 < *P* ≤ 0.05); extremely significant increase (*β* ≥ 0, *P* ≤ 0.01); insignificant decrease (*β* < 0, *P* > 0.05); significant decrease (*β* < 0, 0.01 < *P* ≤ 0.05); and extremely significant decrease (*β* < 0, *P* ≤ 0.01).

#### Climate and anthropogenic driver analysis

Changes in the climate have a significant effect on vegetation, and the most significant drivers of vegetation coverage change are precipitation and temperature^32^. Partial and multiple correlation analyses were used to evaluate the relationships between vegetation coverage and climatic factors^33^. Specifically, partial correlation analysis was used to determine the relationships between climate variables and vegetation coverage while controlling the effects of other variables, and t-tests were used to determine the reliability of the partial regression^34^. The formulae for the partial correlation of *PCC*_*xy*,*z*_ were as follows:

2 $$ PCC_{xy,z} = \frac{{r_{xy} - r_{xz} r_{yz} }}{{\sqrt {\left( {1 - r_{xz}^{2} } \right) + \left( {1 - r_{yz}^{2} } \right)} }} $$

3 $$ r_{xy} = \frac{{\mathop \sum \nolimits_{i = 1}^{n} \left[ {\left( {x_{i} - \overline{x}} \right)\left( {y_{i} - \overline{y}} \right)} \right]}}{{\sqrt {\mathop \sum \nolimits_{i = 1}^{n} \left( {x_{i} - \overline{x}} \right)^{2} \mathop \sum \nolimits_{i = 1}^{n} \left( {y_{i} - \overline{y}} \right)^{2} } }} $$

where *PCC*_*xy*,*z*_ represents the partial correlation coefficient between *x* and *y* when the *z* variable is fixed; *r*_*xy*_, *r*_*xz*_ and *r*_*yz*_ are the pairwise correlation coefficients; *n* is the number of years; *x*_*i*_ and *y*_*i*_ are the values of the *x* and *y* variables in the *i*-th year, respectively, and $\overline{x}$ and $\overline{y}$ are the annual averages of *x* and *y*, respectively. The partial correlation between vegetation coverage, precipitation, and temperature were divided into the following six types according to the results of the t-tests: insignificant positive correlation (*PCC*_*xy*,*z*_ ≥ 0, *P* > 0.05); significant positive correlation (*PCC*_*xy*,*z*_ ≥ 0, 0.01 < *P* ≤ 0.05); extremely significant positive correlation (*PCC*_*xy*,*z*_ ≥ 0, *P* ≤ 0.01); insignificant negative correlation (*PCC*_*xy*,*z*_ < 0, *P* > 0.05); significant negative correlation (*PCC*_*xy*,*z*_ < 0, 0.01 < *P* ≤ 0.05); and extremely significant negative correlation (*PCC*_*xy*,*z*_ < 0, *P* ≤ 0.01).

Multiple correlation analysis was used to estimate the combined impact of precipitation and temperature on vegetation coverage as evaluated by the F-test^26^. The formula is as follows:

4 $$ PCC_{x,yz} = \sqrt {1 - \left( {1 - r_{xy}^{2} } \right)\left( {1 - R_{xz,y}^{2} } \right)} $$

To analyse the impacts of human drivers, a multiple linear regression model was first constructed between precipitation, temperature, and the true vegetation coverage (i.e., the vegetation coverage calculated from the remote sensing datasets). This model was then used to obtain the potential vegetation coverage (i.e., predicted fractional vegetation coverage, FVC) for annual precipitation and temperature. Residual analysis was then used to determine the impact of human activities on vegetation coverage. The general expression for a multiple linear regression model is^35^:

5 $$ y = \beta_{0} + \beta_{1} x_{1} + \beta_{2} x_{2} + \cdots + \beta_{k} x_{k} + \sigma $$

where *β*_0_, *β*_1_, *β*_2_ … *β*_*k*_ are the model’s parameters and *σ* is the error term. This shows that *y* is the sum of the linear function of *x*_1_, *x*_2_, …, *x*_*k*_ and a certain error (*σ*).

Residual analysis was used to separate the effects of human activities and climate change on vegetation coverage based on the differences between the true and predicted vegetation coverage^36^. This approach is founded on two assumptions. First, the dynamics of vegetation coverage are assumed to be mainly dependent on climate and human activities, and second, precipitation and temperature are assumed to be sufficient to characterise the effects of climate on vegetation coverage. The governing formula of residual analysis is as follows:

6 $$ \upvarepsilon = {\text{FVC}}_{{{\text{True}}}} - {\text{FVC}}_{{{\text{Predicted}}}} $$

where ε is the FVC residual; FVC_True_ is the true FVC; and FVC_Predicted_ is the predicted FVC obtained from a multiple linear regression between precipitation, temperature, and the true FVC values. Trend analyses were then performed on the FVC residuals (Eq. (1)). Where *β* > 0, human activities were considered to have a positive impact on vegetation coverage; where *β* < 0, human activities were considered to have a negative impact on vegetation coverage; and where *β* ≈ 0, human activities were considered not to have a significant impact on vegetation coverage.

#### Relative contribution analysis

The vegetation coverage trends associated with climate change and human activities were divided into increasing and decreasing trends to facilitate an analysis of their relative contributions^20^. In this context, ‘relative contribution’ refers to the combined effect of climate change and human activities rather than the relative contributions of each. Details of this analysis are shown in Table 1.

**Table 1:** Relative contributions of climate change and human activities to vegetation coverage trends under different scenarios.

Vegetation coverage trend	Predicted trend	Trend of the residual	Relative contribution of climate change (%)	Relative contribution of human activities (%)	Explanation
> 0	> 0	> 0	$\frac{{\left| {{\text{FVC}}_{{{\text{Predicted}}}} } \right|}}{{\left| {{\text{FVC}}_{{{\text{Predicted}}}} } \right| + \left| \upvarepsilon \right|}}$	$\frac{\left| \upvarepsilon \right|}{{\left| {{\text{FVC}}_{{{\text{Predicted}}}} } \right| + \left| \upvarepsilon \right|}}$	Climate change and human activities both increased vegetation coverage
	> 0	< 0	100	0	Climate change increased vegetation coverage
	< 0	> 0	0	100	Human activities increased vegetation coverage
< 0	< 0	< 0	$\frac{{\left| {{\text{FVC}}_{{{\text{Predicted}}}} } \right|}}{{\left| {{\text{FVC}}_{{{\text{Predicted}}}} } \right| + \left| \upvarepsilon \right|}}$	$\frac{\left| \upvarepsilon \right|}{{\left| {{\text{FVC}}_{{{\text{Predicted}}}} } \right| + \left| \upvarepsilon \right|}}$	Climate change and human activities both decreased vegetation coverage
	< 0	> 0	100	0	Climate change decreased vegetation coverage
	> 0	< 0	0	100	Human activities decreased vegetation coverage

## Results

### Spatial–temporal variations of greening responded by vegetation coverage in the BTHR

**Figure 2:**
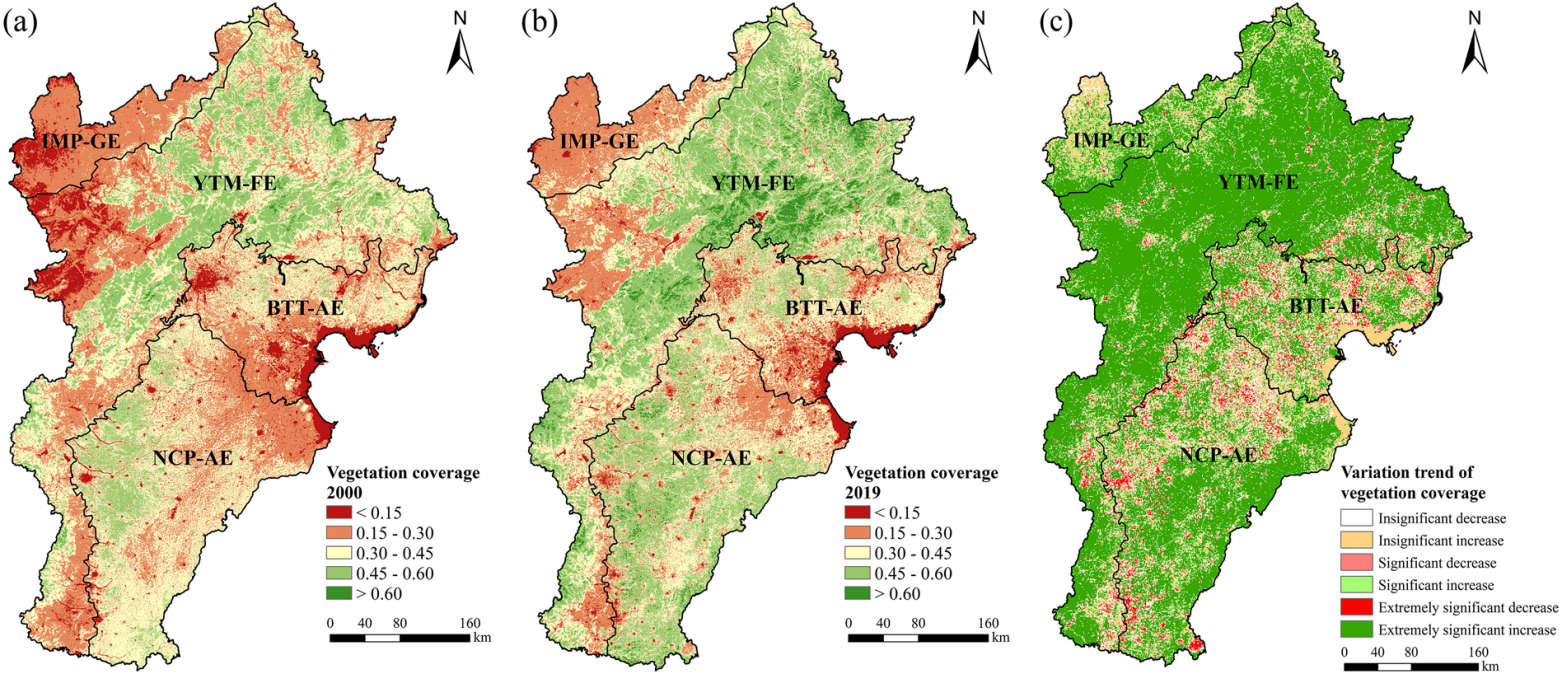
Annual average vegetation coverage in 2000 (**a**) and 2019 (**b**), and spatiotemporal variation trends in the BTHR between 2000 and 2019 (**c**). Map created in ArcMap 10.4.

From 2000 to 2019, the vegetation coverage of the BTHR increased significantly at a rate of 0.036 decade^−1^ (*P* < 0.01). Some small variations occurred within the overall trend, including a fluctuating growth rate of 0.041 decade^−1^ (*P* = 0.011) between 2000 and 2009, and a steady increase of 0.044 decade^−1^ (*P* < 0.01) between 2009 and 2019. Spatial distribution of annual average vegetation coverage in 2000 and 2019 were shown in Fig. 2a,b, respectively.

Spatially, 85.5% of the study area exhibited an increase in vegetation coverage during the study period. The intermontane basin forest-agricultural-grassland zone of the Upper Yongding River in the forest ecoregion and southeast part of the NCP-AE showed high rates increasing vegetation coverage. Significant or extremely significant increases in vegetation coverage were occurred in 67.1% of the study area, with these areas widely distributed across the mountainous and hilly areas of northwest Hebei and the southern part of the south-central Hebei Plains. In contrast, vegetation coverage decreased in 14.5% of the study area, with 5.7% showing significant or extremely significant decreases. These areas include the suburban agricultural zone in the BTT-AE, the northern part of the NCP-AE, and other urban areas (Fig. 2c).

### Effects of climate change on regional greening

#### Climate change between 2000 and 2019

**Figure 3:**
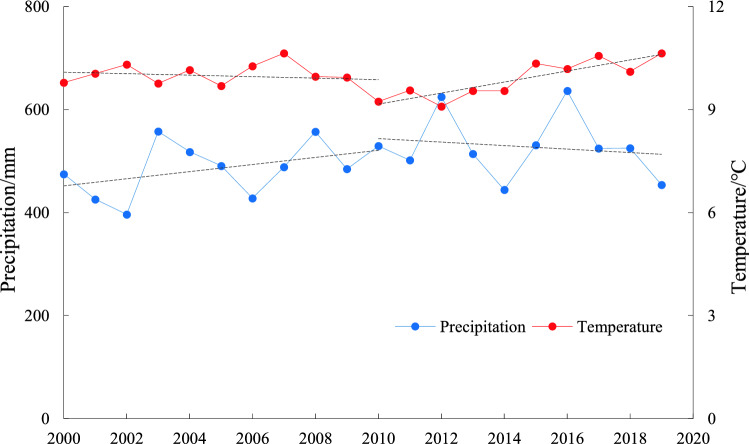
Annual average precipitation and temperature in the BTHR between 2000 and 2019.

Overall, precipitation increased at a rate of 38.45 mm decade^−1^ (*P* = 0.107) between 2000 and 2019, including periods of increase (2000–2010, 69.03 mm decade^−1^, *P* = 0.186) and decrease (2010–2019, 33.88 mm decade^−1^, *P* = 0.647). Temperatures also increased overall between 2000 and 2019 at a rate of 0.096 °C decade^−1^ (*P* = 0.591), including a decrease of 0.22 °C decade^−1^ from 2000 to 2010 (*P* = 0.569) and an increase of 1.61 °C decade^−1^ from 2010 to 2019 (*P* = 0.087) (Fig. 3).

**Figure 4:**
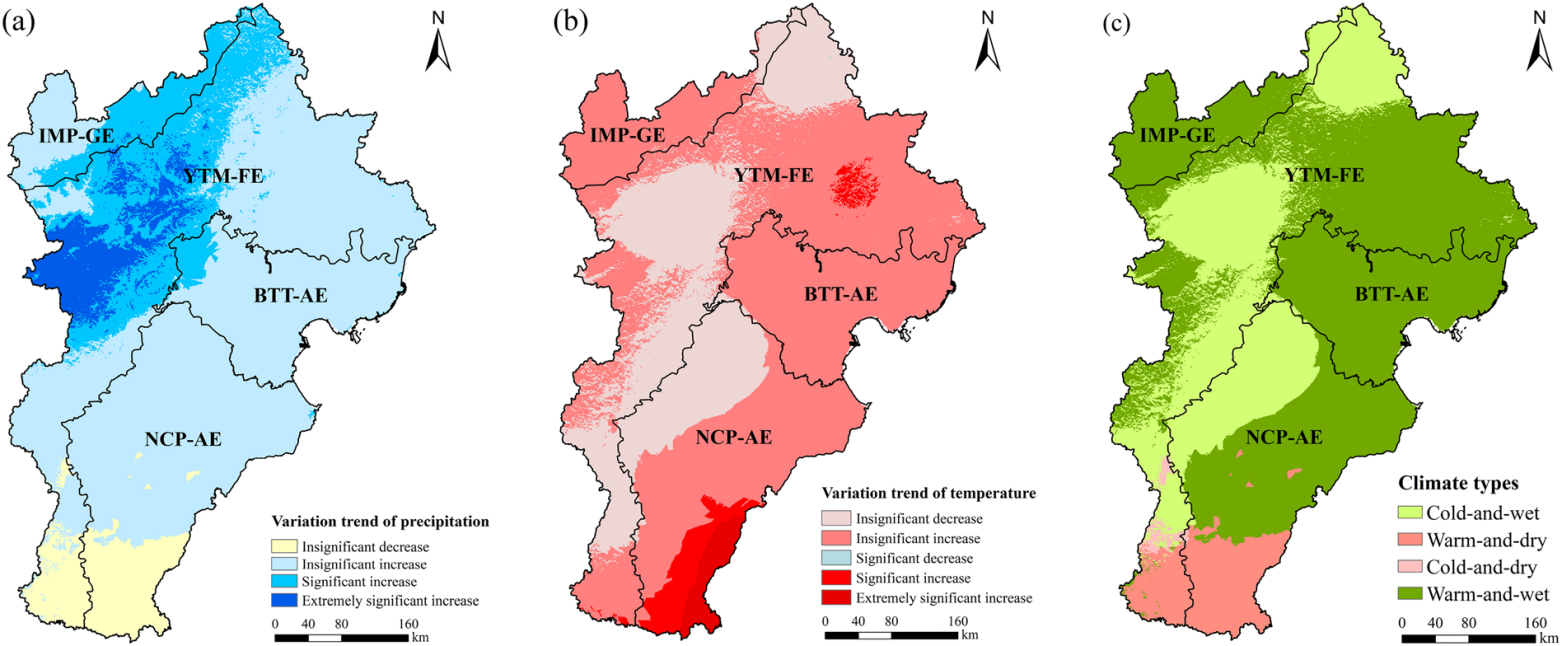
Variation trends of precipitation (**a**), temperature (**b**) and climate types (**c**) in the BTHR between 2000 and 2019. Map created in ArcMap 10.4.

The precipitation trends in the BTHR varied significantly spatially, increasing in the central and northern regions but decreasing in the southern region. Rainfall increased significantly or extremely significantly mainly in the grassland ecoregion, the intermontane basin forest-agricultural-grassland zone of the forest ecoregion, and in central Beijing in the BTT-AE (Fig. 4a). The temperature trends also varied in the east–west direction, increasing and decreasing over time in the eastern and western regions, respectively. The areas that exhibited significant or extremely significant increases in temperature are mainly located in the southeast corner of the NCP-AE (Fig. 4b). Based on these trends, 0.7%, 7.8%, 30.1%, and 61.4% of the study area can be classified as cold-and-dry, warm-and-dry, cold-and-wet, and warm-and-wet areas, respectively (Fig. 4c).

#### Impacts of climate change on regional greening

**Figure 5:**
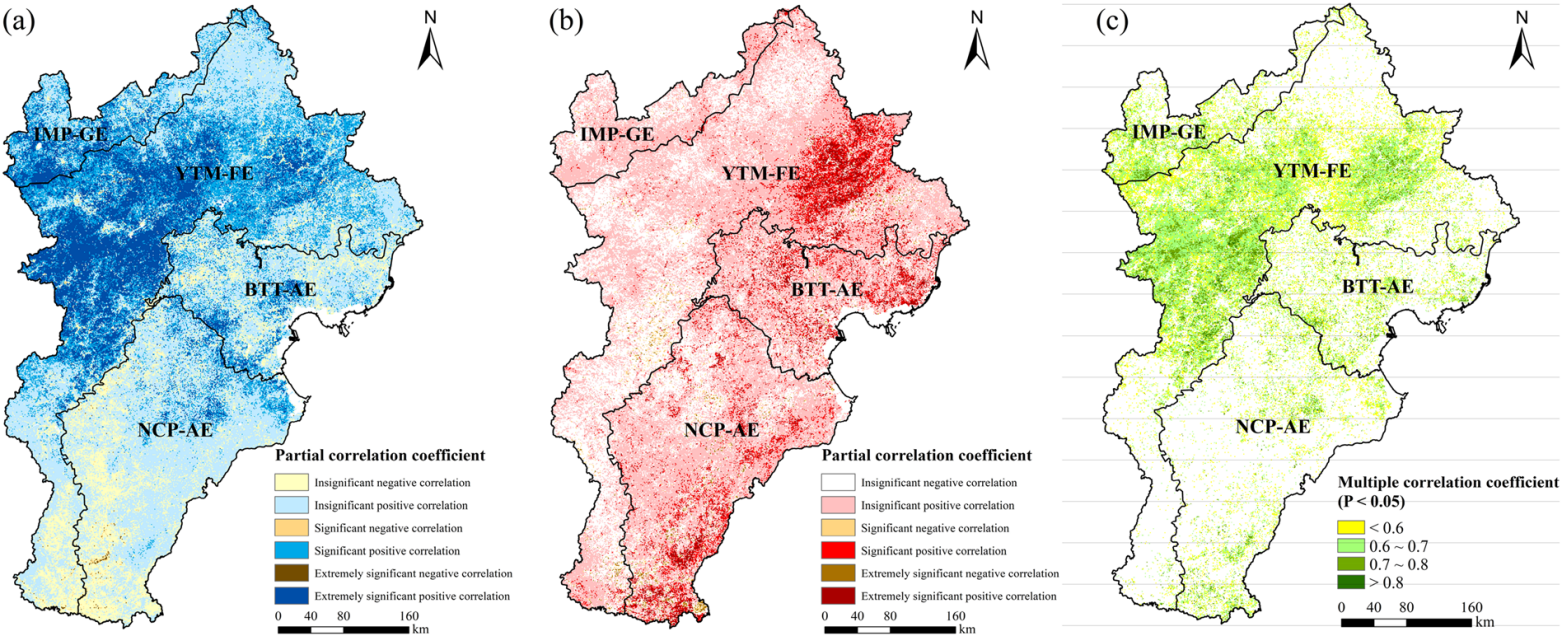
Correlation coefficients between vegetation coverage and precipitation (**a**), temperature (**b**) and both (**c**) in the BTHR between 2000 and 2019. Map created in ArcMap 10.4.

During 2000–2019, vegetation coverage in the BTHR was positively correlated with precipitation (*PCC* = 0.541, *P* = 0.017). Overall, 85.0% of the study area exhibited positive correlations between these two variables, with 37.8% showing significant or extremely significant positive correlations. These areas generally coincided with the areas that experienced significant/extremely significant increases in precipitation. In contrast, 15.0% of the study area showed a negative correlation between precipitation and vegetation coverage, particularly at the boundary between the YTM-FE and the NCP-AE (Fig. 5a).

Vegetation coverage was positively correlated with temperature overall (*PCC* = 0.324, *P* = 0 0.176), with 66.7% of the study area showing positive correlations and 10.6% showing significant or extremely significant positive correlations. These areas were mainly located in the eastern part of the YTM-FE and some parts of the BTT-AE and NCP-AE. A negative correlation between vegetation coverage and temperature was observed in 33.3% of the study area, mostly in the forest ecoregion including the intermontane basin forest-agricultural-grassland zone, the forest zone of the Taihang Mountains, and the Saihanba region (Fig. 5b).

The multiple correlation coefficient between vegetation coverage, precipitation, and temperature (0.552) was also significant (*P* = 0.046), with 32.9% of the study area exhibiting significant correlations (*P* < 0.05). These areas mainly correspond to the IMP-GE and midwest of the YTM-FE (Fig. 5c).

### Human impacts on regional greening

#### Spatiotemporal variations of vegetation coverage residuals

**Figure 6:**
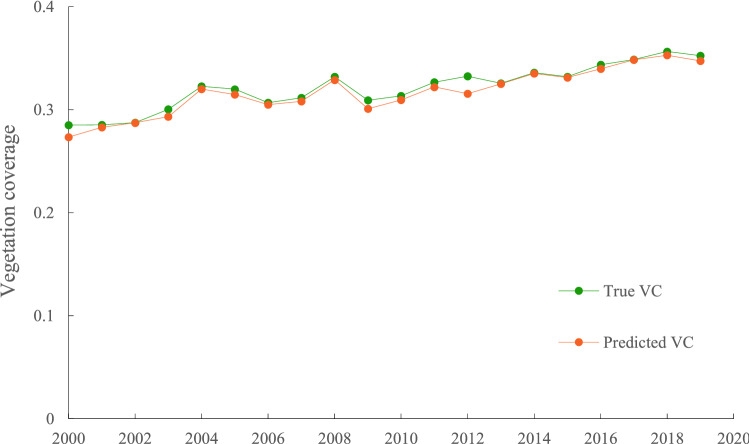
Annual average predicted and true vegetation coverage in the BTHR between 2000 and 2019.

**Figure 7:**
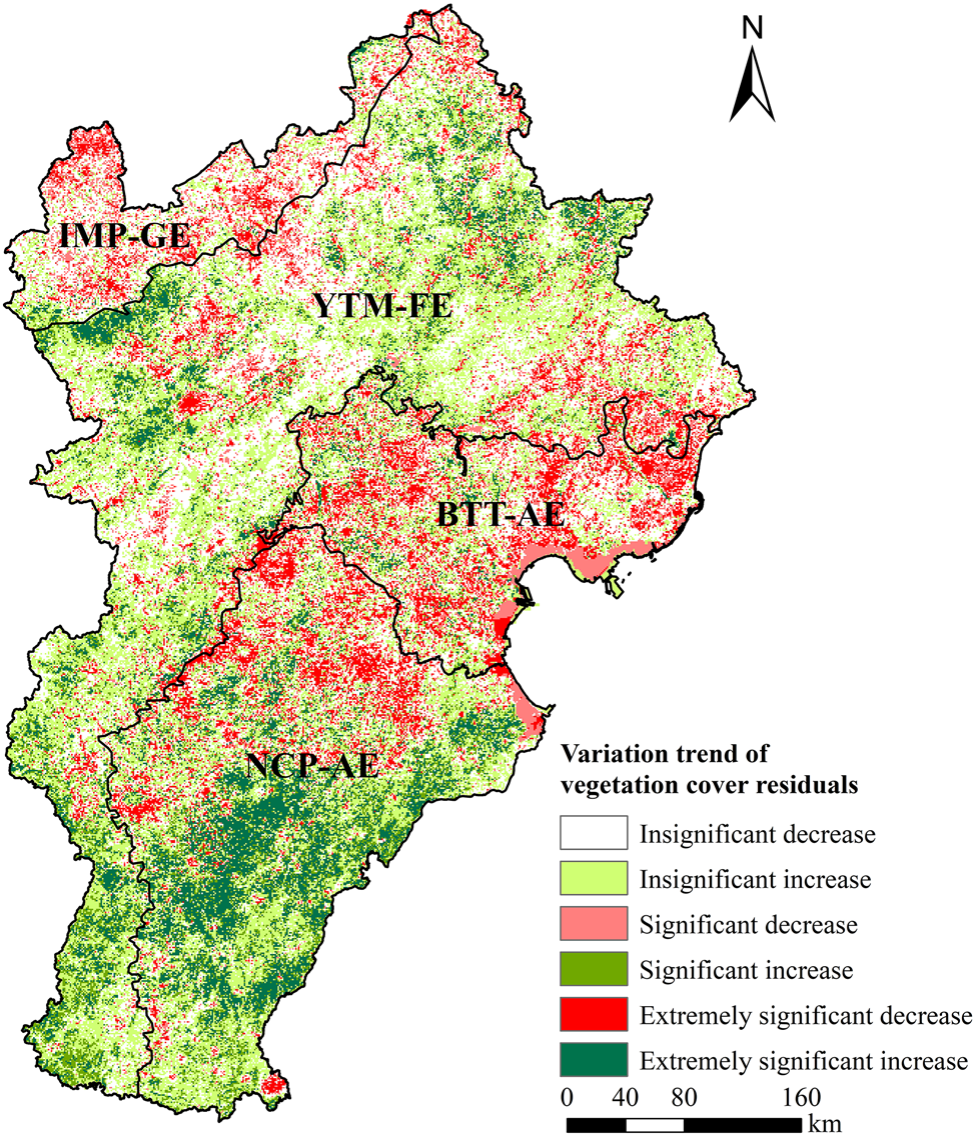
Variation trend of vegetation coverage residuals in the BTHR between 2000 and 2019. Map created in ArcMap 10.4.

The vegetation coverage trends predicted using multiple regression based on precipitation and temperature observations largely agreed with the true vegetation coverage trends in the BTHR. The R^2^ values of the annual regression models were generally greater than 0.7 (*P* < 0.01) (Fig. 6). According to the varying trend of vegetation coverage residuals between 2000 and 2019, human activities had a positive impact on vegetation coverage in 48.4% of the study area, and this (positive) impact was significant/extremely significant in 16.9% of the study area. These areas mainly correspond to the YTM-FE (the mountainous forest zone of north Hebei, the Yanshan and Taihang Mountains, and the intermontane basin forest-agricultural-grassland zone), as well as coastal and central-south of the NCP-AE. In contrast, human activities negatively affected vegetation coverage in 51.6% of the study area, with vegetation coverage significantly/extremely significantly negatively affected by human activities in 18.8% of the study area. These areas mainly correspond to the IMP-GE, the BTT-AE, and the northern part of the NCP-AE (Fig. 7).

#### Impacts of human activities on regional greening induced from land use changes

In 2020, the primary land use types in the BTHR were farmland, forested land, grassland, and artificial surfaces, which occupied 48.34%, 19.48%, 17.69%, and 12.24% of the study area, respectively. From 2000 to 2020, farmland decreased by 10.23% (1.19 × 10^4^ km^2^), artificial surfaces increased by 77.00% (1.15 × 10^4^ km^2^), and 1.15 × 10^4^ km^2^ was converted from farmland to artificial surfaces in association with urbanisation. Forested land increased by 0.82% (341.99 km^2^) while grassland increased by 0.98% (369.17 km^2^). The increases in the forested land and grassland areas were mainly caused by farmland conversion.

Ecological rehabilitation programs in the study region are characterised by the conversion of farmland into forested land and grassland. For example, between 2000 and 2020, 4 402.23 km^2^ of farmland was converted into forests and grassland, increasing the vegetation coverage of these areas as a result. Furthermore, vegetation coverage increased more quickly where conversion was made into forests compared to conversion into grasslands. Therefore, conversion from farmland to forested land is the most effective strategy for regional greening. As another form of human-driven conversion, urban expansion is characterised by the conversion of farmland, forested land, and grassland into artificial surfaces. Over the past 20 years, the scale of urban expansion (associated with the depletion of natural resources) was 12 613.53 km^2^ in the BTHR. The average change in vegetation coverage residual in these areas was always negative, showing that urban expansion has had significant negative impacts on vegetation coverage in this region.

### Relative contributions of climate change and human activities on greening

**Figure 8:**
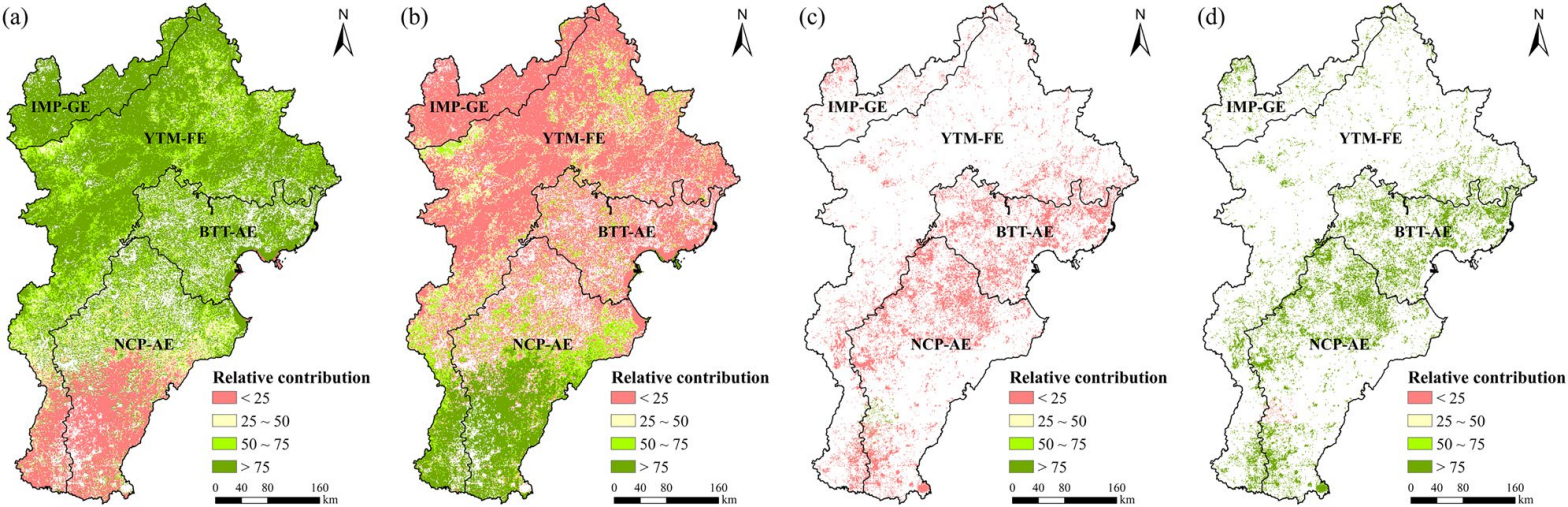
Relative contributions of climate change and human activities to vegetation coverage in the BTHR between 2000 and 2019. Map created in ArcMap 10.4.

According to the relative contribution analysis, climate change and human activities both had positive effects on vegetation coverage in the areas where vegetation greening, with an average relative contribution from climate change of 74.1% (Fig. 8a); the areas where the relative contribution from climate change was > 50% accounted for 77.0% of all areas experiencing increases in vegetation coverage, which were mainly located in the northern and central parts of the study area. In comparison, the average relative contribution of human activities to the areas that experienced vegetation coverage increases was 25.9% (Fig. 8b). Increases in vegetation coverage in the northern and central parts of the study area were primarily caused by climate change, whereas increases in the southern part of the study area were dominated by human activities. In those areas where vegetation browning, the relative contribution of climate change was typically < 20% (Fig. 8c) compared to > 75% (Fig. 8d) for human activities. Therefore, overall, human activity has been the primary driver of the decreases in vegetation coverage in the study area over the last 20 years.

## Discussion

### Driving factors of regional greening

The increase in vegetation coverage in the BTHR over the last 20 years has resulted from the combined influences of climate change and human activities. With respect to climate, areas with warm-and-wet and cold-and-wet climates cover 61.4% and 30.1% of the BTHR, respectively, and the climate-vegetation coverage correlations strongly indicate that increases in precipitation and temperature have been conducive to increases in vegetation coverage. Furthermore, the impact of precipitation on vegetation coverage has been greater than that of temperature—that is, precipitation was found to be significantly positively correlated with vegetation coverage in a larger area compared to the correlations with temperature. The relative contribution analysis also showed that climate change contributed most strongly to increases in vegetation coverage in warm-and-wet and cold-and-wet regions.

Alongside climate impacts, the influences of human activities on regional greening cannot be overlooked. Several rehabilitation programs have been implemented in the BTHR since 2000, including the Beijing–Tianjin Sandstorm Source Control Project, the Taihang Mountain Greening Project, the Plain Afforestation Project, and the Grain for Green Project^37–39^. These initiatives have increased vegetation greening through measures including afforestation, small watershed management, and grassland restoration^40–43^. In addition, changes in the vegetation coverage of agricultural zones can be a direct result of crop farming^44^. The crops planted in the North China Plain are mainly winter wheat, summer maize, and cotton^45^. Although the area covered by food crops in the BTHR decreased from 8 076.8 × 10^3^ hm^2^ in 2000 to 6 944.5 × 10^3^ hm^2^ in 2019, food production increased from 2 819.3 × 10^4^ t to 3 944.7 × 10^4^ t, which corresponds to a rate of change of 78.8 × 10^4^ t decade^−1^ (*P* < 0.01). The increase in vegetation coverage was significantly correlated with increases in food production during this period (*r* = 0.87, *P* < 0.01). Therefore, agricultural activities partly explain the observed increases in vegetation coverage in agricultural zones.

Vegetation coverage decreases in the BTHR have been mainly caused by human activities^25^. For example, the population density increased from 419 to 521 persons/km^2^, the gross regional production increased from 1 101. 8 to 7 164.5 billion CNY, and construction land area (towns, residential land in farming villages, and other types of construction) increased from 22,394.9 to 27,624.6 km^2^ between 2000 and 2019, respectively. Thus, population growth, economic development, and urban expansion in association with urbanisation has had negative environmental impacts in the BTHR, often in association with environmental degradation via the extraction and consumption of natural resources^46^.

### Suggestions for sustainable land use management

Land use changes have direct impacts on regional greening. From 2000 to 2020, the area of returning farmland to forest land and grassland in the BTHR was 1105.66 and 3296.57 km^2^, respectively; while the changing trend of vegetation coverage residuals was 0.00305/10a and 0.00723/10a, respectively. In the meantime, the area of urban expansion characterized by consumption of natural resources in the BTHR had reached 12,613.53 km^2^, and the changing trend of vegetation coverage residuals was -0.019/10a, which indicated that the phenomenon of urban expansion had an obvious inhibiting effect on greening. Thus, sustainable land use management is necessary to maintain and consolidate the improvements on greening in the BTHR in future. On the one hand, continue to implement greening projects compatible with regional natural conditions in order to increase green areas; on the other hand, strengthen ecological protection and restoration is suggested in order to improve the quality and stability of natural ecosystems.

### Limitations and uncertainties

Finally, the following limitations and uncertainties should be acknowledged. First, data on ecological rehabilitation projects, agricultural output, and urbanisation were only available at a coarse scale (i.e., municipal-level data). Therefore, to determine the primary mode by which human activities affect regional greening, field survey data and higher-resolution remote sensing data are required for some regions. More detailed analyses of socioeconomic statistics are also required. Second, using multiple linear regression to predict vegetation coverage inevitably overlooks the nonlinear responses of vegetation to climate change as well as any time lags and cumulative effects on vegetation growth^47–49^. These effects increase the magnitude of errors in driving factor analyses, which would benefit from further consideration.

## Conclusions

The spatiotemporal trends of greening responded by vegetation coverage in the BTHR from 2000 to 2019 and their responses to climate change and human activities were analysed and evaluated. Based on this, the following conclusions can be drawn: (1) vegetation coverage has generally increased over the last 20 years at an average rate of 0.036 decade^−1^ (*P* < 0.01). This increase has occurred across large swathes of the BTHR, with areas showing decreases in vegetation coverage mainly located in cities, townships, and suburban agricultural zones; (2) the climate has become warmer and wetter overall across the study region, with an increased rate of 0.096 °C decade^−1^ and 38.45 mm decade^−1^, respectively. Significant/extremely significant increases in both precipitation and temperature correlate significantly/extremely significantly with increases in vegetation coverage, nearly 32.9% of the study area exhibited significant correlations (*P* < 0.05). Thus, climate changes over the last 20 years have been conducive to widespread increases in vegetation coverage in the BTHR; (3) areas where human activities have increased vegetation coverage are mainly located in the forest ecoregion (the mountainous forest zone of north Hebei, the Yanshan and Taihang Mountains, and the intermontane basin forest-agricultural-grassland zone) as well as the coastal and central-south areas of the NCP-AE. The areas where human activities have reduced vegetation coverage are mainly located in the IMP-GE, the BTT-AE, and the northern part of the NCP-AE; and (4) increases in vegetation coverage have resulted from the combined effects of climate change and human activities. Specifically, a warmer and wetter climate, agricultural activities, and ecological engineering (e.g., afforestation) have jointly increased vegetation coverage in the BTHR. However, climate change has had a stronger influence on vegetation coverage than human activities. Contributions of climate change to greening and browning in the BTHR was about 74.1% and < 20%, respectively. Notably, urbanisation-related phenomena including population growth, rapid economic development, and the expansion of construction land have had negative effects on regional greening.
